# Supplementary material for: Face selective patches in marmoset frontal cortex
Source: Nat Commun. 2020 Sep 25;11:4856. doi: 10.1038/s41467-020-18692-2 (PMC7519082; doi:10.1038/s41467-020-18692-2)
Supplement: Supplementary file 2 — Reporting Summary [file 41467_2020_18692_MOESM2_ESM.pdf]

nature research

Corresponding author(s): David J. Schaeffer, Stefan Everling

Last updated by author(s): Aug 25, 2020

Reporting Summary

Nature Research wishes to improve the reproducibility of the work that we publish. This form provides structure for consistency and transparency in reporting. For further information on Nature Research policies, see our Editorial Policies and the Editorial Policy Checklist.

Statistics

For all statistical analyses, confirm that the following items are present in the figure legend, table legend, main text, or Methods section.

n/a	Confirmed	
☐	☒	The exact sample size (*n*) for each experimental group/condition, given as a discrete number and unit of measurement
☐	☒	A statement on whether measurements were taken from distinct samples or whether the same sample was measured repeatedly
☐	☒	The statistical test(s) used AND whether they are one- or two-sided<br>*Only common tests should be described solely by name; describe more complex techniques in the Methods section.*
☐	☒	A description of all covariates tested
☒	☐	A description of any assumptions or corrections, such as tests of normality and adjustment for multiple comparisons
☐	☒	A full description of the statistical parameters including central tendency (e.g. means) or other basic estimates (e.g. regression coefficient) AND variation (e.g. standard deviation) or associated estimates of uncertainty (e.g. confidence intervals)
☐	☒	For null hypothesis testing, the test statistic (e.g. *F*, *t*, *r*) with confidence intervals, effect sizes, degrees of freedom and *P* value noted<br>*Give P values as exact values whenever suitable.*
☒	☐	For Bayesian analysis, information on the choice of priors and Markov chain Monte Carlo settings
☒	☐	For hierarchical and complex designs, identification of the appropriate level for tests and full reporting of outcomes
☐	☒	Estimates of effect sizes (e.g. Cohen's *d*, Pearson's *r*), indicating how they were calculated

Our web collection on statistics for biologists contains articles on many of the points above.

Software and code

Policy information about availability of computer code

Data collection	Python 2.7.16, CORTEX, SPSS (v.25, IBM Corp, 2019), Paravision-6
Data analysis	FMRIB Software Library v6.0<br>AFNI Version AFNI_20.0.19 'Galba'<br>ANTs Version 5<br>Matlab 2019b<br>Python 2.7.16 - custom code for eye tracking analysis available at https://gin.g-node.org/everling_lab_marmosets/marmoset_face_processing

For manuscripts utilizing custom algorithms or software that are central to the research but not yet described in published literature, software must be made available to editors and reviewers. We strongly encourage code deposition in a community repository (e.g. GitHub). See the Nature Research guidelines for submitting code & software for further information.

Data

Policy information about availability of data

All manuscripts must include a data availability statement. This statement should provide the following information, where applicable:

- Accession codes, unique identifiers, or web links for publicly available datasets
- A list of figures that have associated raw data
- A description of any restrictions on data availability

The datasets generated during and/or analyzed during the current study are available in the https://gin.g-node.org/ repository, https://gin.g-node.org/everling_lab_marmosets/marmoset_face_processing.

Field-specific reporting

Please select the one below that is the best fit for your research. If you are not sure, read the appropriate sections before making your selection.

☒ Life sciences ☐ Behavioural & social sciences ☐ Ecological, evolutionary & environmental sciences

For a reference copy of the document with all sections, see nature.com/documents/nr-reporting-summary-flat.pdf

Life sciences study design

All studies must disclose on these points even when the disclosure is negative.

Sample size	Sample size was determined by repeatability of spatial fMRI patterns at the individual animal level, with all four marmosets showing comparable topologies at the individual level, suggesting that more animals were not needed given the correspondence in every animal.
Data exclusions	fMRI runs in which animals did not have their eyes open (as assessed by MR compatible camera) were not included - this criteria was pr-established, with the eyes being closed not allowing the monkeys see the stimuli and thus not evoking the topology of interest.
Replication	Group results were repeatable at the individual animal level, with face patches visible (albiet less robust) in every individual fMRI run.
Randomization	All animals participated in both experimental and control conditions, for which 3 stimulus sets were used (counterbalanced between animals), with four pseudo-randomized task conditions each (directed gaze, averted gaze, and scrambled versions of each).
Blinding	Experimenters were not blinded during the experiment as all experimenters were familiar with each marmoset and could identified them, but identifiers were randomly assigned for analysis and as such, the experimenters were blind to the specific animal at the point of analysis.

Reporting for specific materials, systems and methods

We require information from authors about some types of materials, experimental systems and methods used in many studies. Here, indicate whether each material, system or method listed is relevant to your study. If you are not sure if a list item applies to your research, read the appropriate section before selecting a response.

Materials & experimental systems

n/a	Involved in the study	
☒	☐	Antibodies
☒	☐	Eukaryotic cell lines
☒	☐	Palaeontology and archaeology
☐	☒	Animals and other organisms
☒	☐	Human research participants
☒	☐	Clinical data
☒	☐	Dual use research of concern

Methods

n/a	Involved in the study	
☒	☐	ChIP-seq
☒	☐	Flow cytometry
☐	☒	MRI-based neuroimaging

Animals and other organisms

Policy information about studies involving animals; ARRIVE guidelines recommended for reporting animal research

Laboratory animals	Data were collected from 10 adult marmosets (Callithrix jacchus; three female; age 29 - 74 months).
Wild animals	This study did not involve wild animals.
Field-collected samples	This study did not involve field-collected samples.
Ethics oversight	Experimental procedures were in accordance with the Canadian Council of Animal Care policy and a protocol approved by the Animal Care Committee of the University of Western Ontario Council on Animal Care.

Note that full information on the approval of the study protocol must also be provided in the manuscript.

Magnetic resonance imaging

Experimental design

Design type	Block-design fMRI and resting-state fMRI
Design specifications	A block design was used in which nine baseline blocks (18 sec each) were alternated with eight task blocks (12 sec each)

Behavioral performance measures

Eye tracking was performed outside of the MRI environment using the same task - there eye velocity, fixation duration, and position were calculated.

Acquisition

Imaging type(s)

functional

Field strength

9.4T

Sequence & imaging parameters

Functional imaging was performed over multiple sessions (days) for each animal, with 6 – 8 task-based functional runs (at 172 volumes each) per animal with the following parameters: TR = 1500 ms, TE = 15 ms, flip angle = 40 degrees, field of view = 64 x 64 mm, matrix size = 128 x 128, voxel size = 0.5 x 0.5 x 0.5 mm, slices = 42, bandwidth = 500 kHz, GRAPPA acceleration factor: 2 (anterior-posterior).

Area of acquisition

Whole brain fMRI.

Diffusion MRI ☐ Used ☒ Not used

Preprocessing

Preprocessing software

FMRIB Software Library v6.0
AFNI Version AFNI_20.0.19 'Galba'
ANTs Version 5

The fMRI data was preprocessed using AFNI (Cox, 1996) and FMRIB/FSL (Smith et al., 2004). Raw functional images were converted to NifTI format using dcm2niix (Li et al., 2016) and reoriented from the sphinx position using FSL. The images were then despiked (AFNI's 3dDespike) and volume registered to the middle volume of each time series (AFNI's 3dvolreg). The motion parameters from volume registration were stored for later use with nuisance regression. Images were smoothed by a 1.5 mm full-width at half-maximum Gaussian kernel to reduce noise (AFNI's 3dmerge). An average functional image was then calculated for each session and registered (FSL's FLIRT) to each animal's T2-weighted image – the 4D time series data was carried over using this transformation matrix. Anatomical images were manually skull-stripped and this mask was applied to the functional images in anatomical space. The T2-weighted images were then non-linearly registered to the NIH marmoset brain atlas (Liu et al., 2018) using Advanced Normalization Tools (ANTs; Avants et al., 2011) and the resultant transformation matrices stored for later transformation (see below). The olfactory bulb was manually removed from the T2-weighted images of each animal prior to registration, as it was not included in the template image.

Normalization

FSL's FLIRT (12 parameter), then non-linear transformation using Advanced Normalization Tools

Normalization template

NIH Marmoset Brain Atlas (Version 1)

Noise and artifact removal

See above.

Volume censoring

Volumes were censored via the scanner console before raw images were saved.

Statistical modeling & inference

Model type and settings

3dDeconvolve (Ordinary Least Squares (OLSQ) regression) with each task condition as an input variable, then noise regressors for head motion, linear and nonlinear trends (X ^ 5).

Effect(s) tested

All task conditions (directed gaze, averted gaze, scrambled versions of each) were entered into the model and convolved with the hemodynamic response (AFNI's 'BLOCK' convolution) - residual effects were output for each task condition.

Specify type of analysis: ☐ Whole brain ☐ ROI-based ☒ Both

Anatomical location(s)

Locations for seed analysis were determined by peaks provided from the task-based analysis.

Statistic type for inference
(See Eklund et al. 2016)

Voxel-wise

Correction

Monte Carlo

Models & analysis

n/a	Involved in the study
☐	☒ Functional and/or effective connectivity
☒	☐ Graph analysis
☒	☐ Multivariate modeling or predictive analysis

Functional and/or effective connectivity

Pearson correlation.
